# Prevention of internalizing disorders in 9–10 year old children: efficacy of the Aussie Optimism Positive Thinking Skills Program at 30-month follow-up

**DOI:** 10.3389/fpsyg.2013.00988

**Published:** 2013-12-26

**Authors:** Rosanna M. Rooney, David Morrison, Sharinaz Hassan, Robert Kane, Clare Roberts, Vincent Mancini

**Affiliations:** Faculty of Health Sciences, School of Psychology and Speech Pathology, Curtin UniversityPerth, WA, Australia

**Keywords:** primary school, internalizing disorders, externalizing behaviors, intervention programs for children, Aussie Optimism, medium-term follow up

## Abstract

The Aussie Optimism: Positive Thinking Skills Program (AOPTP) is a school-based prevention program aimed at addressing anxious and depressive symptoms in children aged 9–10 years. Nine-hundred and ten students from 22 Australian primary schools situated in low socio-economic areas were randomly assigned to either an intervention or control group, and assessed at a 30-month follow up. Those in the intervention group received the AOPTP program, where the control group continued to receive the regular health education course. Students completed self-report measures regarding their levels of depression, anxiety, and attribution style. Parents also reported on their children's externalizing and internalizing problems outside of school. There were no significant differences between groups in regard to anxiety or depression, as well as no significant differences in attributional styles. Parents reported significantly less hyperactive behaviors from children in the intervention group. This finding suggests that AOP-PTS has the capacity to treat externalizing problems at a medium term effect. The decrease in the externalizing problems provides evidence of a partial medium term intervention effect. Future studies should continue to evaluate the program at a long term follow up.

## Introduction

The psychological adversities commonly experienced by children and adolescents are often classified into one of two broad categories: externalizing disorders, and internalizing disorders. Though conceptually different, these disorders are not always independent from one another, and have the capacity to impact an individual in conjunction. Originally introduced by Achenbach and Edelbrock (1978), these terms were used to differentiate the different psychopathologies pertinent to each category. Externalizing disorders are characterized by disruptive, disobedient, and harmful behaviors that are often manifested physically (Weisz and Weiss, 1991), such as aggressive, impulsive, and non-compliant actions (Achenbach, 1991). Comparatively, internalizing disorders are characterized through feelings of sorry, guilt, worry, and somatization (Weisz and Weiss, 1991), and expressed through actions such as social withdrawal, a lack of pleasure from enjoyable activities, and a lack of energy (Cicchetti and Toth, 1998). The most prevalent internalizing disorders in childhood and adolescence are anxiety and mood disorders. Subsequently, these disorders are often the primary focus for mental health prevention programs that aim to address the issue of internalizing disorders. In Australia, anxiety and depression are the most common mental health issues experienced by children and adolescents (Stanley, 2002). The Australian Mental Health and Behavior Problems survey in 2004–2005 reported that 9% of Australian children below the age of 14 years experienced anxiety related problems, and 3% experienced mood affected problems (Australian Bureau of Statistics, 2006). It is predicted that at any time, 14% of Australian children and adolescents between 4 and 17 years of age experience these internalizing disorders (Sawyer et al., 2001).

Anxiety disorders have the capacity to severely impact on the well-being of a child or adolescent, and are associated with extensive, personal, social, and academic detriments (Neil and Christensen, 2009), which place a very large burden on the individual and the wider community. The development of anxiety disorders also predisposes the individual to the development of other psychological disorders, including depression. The link between anxiety and depression in both childhood and adolescence is extensively supported (Kendall et al., 1992; Cole et al., 1998), with previous research examining the rate of comorbidity between anxiety and depression to be as high as 60% (Avenevoli et al., 2001). Children with comorbid anxiety and depression report more severe symptoms of both disorders, and are rated by clinicians to be more impaired that children suffering from either anxiety or depression in isolation (Manassis and Menna, 1999). Additionally, young adults with a childhood history of comorbid anxiety and depression are less likely to be employed (or in school), more likely to use mental health services, and often report ongoing psychological problems compared to young adults with a childhood history of either anxiety or depression alone (Last et al., 1997).

There are both risks and protective factors that are associated with the development of childhood mental health disorders. Factors that have been consistently linked with the development of childhood depression include the parental history of depression (Diego et al., 2001), and negative life events (Ge et al., 1994; Kessler, 1997). Other identified risk factors include problematic peer relationships (Garland and Fitzgerald, 1998) and negative parental behavior (Lau and Kwok, 2000). The exposure to traumatic events and to negative social outcomes (such as lack of social connectedness and alienation from others) may increase the risk of the development of a childhood anxiety disorder (Barlow, 2008). Family dynamics and their psychological influence may also facilitate the risk of development or childhood anxiety, with studies showing a strong correlation between a child and their parent's levels of fear and dread (Capps et al., 2006).

Protective factors that may protect children against developing depressive symptoms include: high levels of intelligence, good problem solving abilities, and good social skills (Conrad and Hammen, 1993). Children who are high in self-esteem, high in levels of perceived control, and who have positive expectations of the future are less likely to develop depressive symptoms in the presence of environmental risk factors (Herman-Stahl and Petersen, 1999). A positive attribution style has also been examined to be a protective factor against the onset of depressive symptoms (Abramson, 2000). Social support, for example good peer relations and teacher support (Cheung, 1995), and a warm, stable relationship with at least one parent (Herman-Stahl and Petersen, 1999) have also been examined to be factors that protect the child from the onset of childhood depression. Similarly, family support has been examined to be a protecting factor from the development of childhood anxiety (Morris and March, 2004). Other factors that have been examined to protect against the development of childhood anxiety disorders include: regular participation in physical activity, a positive internal locus of control, and a secure attachment style (Freedman et al., 2003). Attachment to family can be classified as protective factor against emotional distress when there is a presence of secure relationship between the parents and the child, whilst the attachment theory posits that the quality of early attachment influences the child's reactions to novel situation, objects, and people (Bowlby, 1971).

Children who experience anxiety and depression tend to experience more cognitive distortions and maladaptive thinking styles compared to children without these disorders (Greenberg et al., 2000). These children exhibit impaired problem solving skills and a pessimistic or irrational cognitive style that impacts their perceptions (Greenberg et al., 2000). They also report feeling a lack of control over their lives and are more likely to have depressive and hostile attributional biases (Kaslow et al., 1994). Anxious children tend to have distorted perceptions of the degree of threat present in certain situations and lack the self-efficacy or effective coping skills to manage their internal distress (Greenberg et al., 2000). Thus, cognitions regarding self-efficacy, self-control, and cognitive distortions have become a focus for prevention programs.

According to Turner and Cole (1994), attribution style appears to mediate rather than moderate the relationship between negative life events and depression in early to middle childhood, which is in contrast to adult populations. This mediating relationship is posited to exist as an individual's thinking styles are still forming during childhood and are not yet stable enough to influence the interpretation of negative life events. Gibb and Alloy (2006) hypothesized that negative life events that occur during early to middle childhood (such as teasing, rejection, and humiliation) may contribute to the formation of a negative thinking style. This negative thinking style exposes the child to an increased vulnerability to depression, where their attribution style mediates, rather than moderates this relationship between negative life events and depression. In their research, Gibb and Alloy (2006) provided partial support for this hypothesis, examining attribution style to have a mediating role in a cohort of Year 4 students. However, the authors found that for slightly older Year 5 students, attribution style was both moderate and mediated the relationship between negative life events and the development of depression.

Childhood is an important period to introduce various emotional competence skills. During childhood, lifelong cognitive skills and thinking styles are still in development. While thinking styles may not be a risk factor for psychopathology, there is evidence that indicates pessimistic or negative attributional styles at this age is related to the development of depression in later adolescence (Nolen-Hoeksema et al., 1992). By facilitating the development of these emotional competence skills in childhood, these skills will act as protective factors in later life.

Research has revealed that early primary school years are the critical stage for students to develop and integrate skills on cognition, emotion, and behavior (Buckley et al., 2003). Emotional competence is essential for the social well-being and positive development of the child. Research has shown that when children experience depression, their negative attribution increases and occasionally remains after the depression subsides, exposing them to an increased risk of developing depression in the future (Nolen-Hoeksema et al., 1992). As such, the middle childhood years of 8–10 years of age represents a critical period in which social competence and emotional skills are developed. Throughout the lifespan, children experience an increasing amount of life events. Exposure to particularly stressful or negative life events exposes the child to developing more permanent and stable pessimistic thinking styles, subsequently increasing the possibility for the development of depression (Cole et al., 2007). The multitude of negative impacts associated with the development of internalizing disorders in childhood expressed a significant need for the development of a childhood prevention program that targets emotional competence skills, negative-self perceptions and cognitive vulnerabilities (Roberts et al., 2010).

Prevention programs which have been used to target anxious and depressive symptoms identified in children have utilized educational institutions like schools as an ideal environment in which programs can be implemented. Schools are viewed as an ideal environment to offer prevention programs as they represent a place where the majority of the population can be reached (Calear and Christensen, 2010). School-based prevention programs are also regarded as effective given their ability to target mental health in children and adolescents who would otherwise be reluctant to seek mental health support from public services. Furthermore, school-based prevention programs have also been observed to reduce the individual disability associated with mental health problems as well as reduce dependency on mental health services (Swannell et al., 2009). School based prevention programs can be offered in universal, selective, and indicated formats. Selective and indicated programs have been examined to produce mixed results in their ability to prevent internalizing disorders in school settings (e.g., Gilliam et al., 1995; Roberts et al., 2004). Selective and indicated programs are designed for individuals who are at high risk for a problem where those in indicated programs are at a higher risk than those in selective programs who have been estimated to only be at average risk for a disorder (Gordon, 1987; Stice et al., 2009). As selected and indicated programs only work with children who have previously been identified as at risk of developing internalizing disorders like anxiety and depression, individuals in the school that were not identified as at risk at the time of testing do not participate in the program, regardless of if their risk of developing these disorders had since increased. These approaches can also lead to social stigma of being selected (Roberts et al., 2008), and may result in embarrassment or shame after attending the programs, which may expose the child or adolescent to a range social and emotional issues.

Alternatively, universal prevention programs are delivered to all children regardless of symptom levels and risk status. This method involves all children in the school age population to receive training on skills needed to protect them from developing psychological disorders in the future. School administrators often prefer universal programs as they do not stigmatize students who may be at risk of developing the internalizing problems with the advantage that no extra time is needed to screen the participants (Calear and Christensen, 2010).

A number of universal school-based prevention programs have shown mixed results in their ability to prevent anxiety and depression amongst children. The Penn Prevention Program (PPP) targets the cognitive distortions of children and aims to improve children's coping skills (Greenberg et al., 2000). The PPP has been used in both selective (Jaycox et al., 1994) and universal (Quayle et al., 2001) programs, and has been shown to be an effective tool in preventing depressive symptoms. The FRIENDS program was found to be effective in reducing anxiety symptoms but only partly effective in reducing depressive symptoms (Barrett and Turner, 2001). The Aussie Optimism: Positive Thinking Program (AOPTP; Rooney et al., 2000) is an alternative universal program that builds on the highly regarded PPP. The AOPTP was specifically designed to prevent anxiety and depression disorders and symptoms, promote positive thinking styles, identify emotional regulation, and foster the development of social competence skills for individuals in the middle stages of childhood (8–10 years).

A pilot study using a randomized controlled trial was conducted with four schools to assess the efficacy of the AOPTP in reducing depressive symptoms (Rooney et al., 2006). Results indicated that the intervention group reported a significant decrease in depressive symptoms and a significant increase in positive attribution at post-test compared to the control conditions. A 9 month follow-up of study indicated that a significantly smaller proportion of the intervention group had since developed a depressive disorder compared to the control condition. These results indicated a prevention effect and suggested that the AOPTP universal prevention program is a potentially effective method to prevent depressive disorders in children as young as 9–10 years of age. Around the same time, research by McLoone et al. (2006) provided evidence suggesting that to effectively treat children with comorbid anxiety and depression, programs should include enactive programming activities. Kendall et al. (1992) suggested that to address co-morbid depressive and anxiety disorders from a cognitive-behavioral theoretical perspective, enactive programming should be included. This would incorporate the construction of a fear hierarchy and exposure to the feared stimulus for anxiety and for depression this would include the scheduling of pleasant events. The inclusion of enactive programming would maximize chances of addressing both types of internalizing disorders.

To foster and enhance further positive intervention effects, the AOPTP has since been modified from its original eight module program format to a 10 session (one session per week for 10 weeks) module that employs enactive programming activities. With reference to the findings provided by McLoone et al. (2006), for anxiety, this involves the construction of fear hierarchy and exposure to a feared object or situation once adequate coping skills have been acquired. To target depression, includes scheduling of pleasant events and incorporating them into our regular routine.

Rooney et al. (2013) conducted a study with the revised version of the AOPTP using a longitudinal randomized controlled trial on 9–10 year old Australian school children. Results showed that the updated AOPTS had significantly reduced depressive symptoms immediately after the program. Parents in the intervention condition reported that their children were less hyperactive after exposure to the program, with this effect observed up to 6-months post-intervention. However, there were no significant effects for reductions in levels of anxiety or for attributional styles. The higher degree of cognitive competence required for the revised AOPTP may have been an influential factor in the formation of these non-significant findings. Since this study, the AOPTP has since been modified to simplify the cognitive component of the program, while also adding an additional emphasis on feelings and empathy skills. It is also possible that any resilience skills acquired by the children in the program may have not had sufficient time to translate into prevention effects. Thus, it is important to determine the long term benefit of the AOPTP by conducting a follow-up with the same cohort of children. Few studies have published the results of long term follow-ups, thus it will be important to determine any prevention effect at 30 month follow-up.

This paper will assess the efficacy of the revised and enhanced AOPTP to prevent anxiety and depressive disorders amongst school children between 9 and 10 years of age, compared to a control group who continue to received their regular health education curriculum, at a 30-month follow up. It is postulated that children involved in the AOPTP will develop significantly fewer depressive and anxiety disorders, and have less pessimistic attribution styles compared to children in the control condition. It is also predicted that parent reports of internalizing and externalizing symptoms will be significantly less in the intervention condition compared to the control condition.

## Methods

### Participants

A total of 910 (89.4%) of the recruited Year 4 students from 22 Australian primary schools were given parental consent to participate in the pre-test phase of this study. Eleven schools were randomly selected from low socioeconomic areas in the Perth metropolitan area, with another 11 schools being matched on potentially confounding variables (e.g., education Department priority rating, school size, and class sizes), giving a total sample of 22 schools. One school from each pair was randomly allocated to the intervention condition (the other school being allocated to the control condition0) such that there were an equal number of schools in each condition. The mean age of participants was 8.75 years of age (*SD* = 0.36 years), with slightly over half of the sample being male (*N* = 467, or 51.4%). A majority of participants were identified as Australian (*N* = 779, or 85.6%), with the remaining participants being identified as culturally and linguistically diverse (CALD). There were 446 students in the intervention group, and 443 in the control group. No significant between groups differences in student age were reported, *F*_(1, 907)_ = 0.882, *p* = 0.348; male/female ratio, χ^2^_(1, *n* = 910)_ = 0.102, *p* = 0.749; or Australian/CALD ratio, χ^2^_(1, *n* = 910)_ = 2.51, *p* = 0.113.

### Measures

#### The children's depression inventory (CDI)

Developed by Kovacs (1992), the CDI is a 27-item, self-rated symptom-oriented instrument for assessing depression in children between the ages of 7–17 years. Children choose from three response choices (reflecting absence of symptom, mild symptom, and definite symptom) to reflect their experience in the last 2 weeks. The CDI Profile presents a total score, plus five empirically developed factors: negative mood; ineffectiveness; negative self-esteem; interpersonal problems, and anhedonia. The CDI has adequate psychometric properties with good internal consistency, with alpha coefficients ranging from 0.71 to 0.89. The CDI also has good convergent validity with clinicians rating of depressive symptomatology based on the Interview Schedule for Children (*r* = 0.55), and CASQ-R composite negative (CN) scores (*r* = 0.55). In this study, the suicide item (9) was omitted from the scale as school principals voiced concern about the use of this item with children as young as eight.

#### Spence children's anxiety scale (SCAS)

The SCAS (Spence, 1994) was developed to assess the severity of anxiety symptoms in line with anxiety disorders proposed by the DSM-IV (American Psychiatric Association, 2000). The SCAS consists of 45 items that measure children's feelings of anxiety on six subscales: generalized anxiety disorder, obsessive compulsive disorder, specific phobia, panic and agroraphobia, separation anxiety, and social anxiety. Participants are asked to rate the description of items on a four-point Likert scale (i.e., never, sometime, often, or always). The SCAS has demonstrated high reliability with alpha coefficient of.92 and test retest reliability of.60. The SCAS has also demonstrated high convergent validity with the Revised Children's Manifest Anxiety Scale (RCMAS) (*r* = 0.73).

#### The children's attributional questionnaire (CASQ)

The CASQ (Seligman et al., 1984) was developed to allow researchers to study attributional styles in children aged 8–13. The CASQ includes 48 items divided equally between positive and negative events. Low composite positive (CP) scores and high CN scores indicate pessimistic explanations. Moderate internal consistency (CP: Cronbach's alpha = 0.53–0.60; CN: Cronbach's alpha = 0.45–0.46) and test-retest reliability (CP: *r* = 0.53; CN: *r* = 0.38) were reported. The CASQ has also demonstrated good convergent validity with the CDI (*r* = 0.46 for CP; *r* = 0.49 CN) and the RCMAS (*r* = 0.41 for CP; *r* = 0.34 for CN).

#### Strength and difficulties questionnaire (SDQ)

The parent version of the SDQ-P (Goodman, 1997) is a 25 item behavioral screening questionnaire measuring parental perceptions of internalizing and externalizing problems for children and adolescents aged 4 through to 16 years. The SDQ-P has good convergent validity with the Connor's Parent Symptom Questionnaire (PSQ) (*r* = 0.63). Internal consistency coefficients of the SDQ-P range between 0.76 and 0.82, and the measure reported a test retest reliability of 0.96.

#### Demographic information

Demographics collected from parents include date of birth, ethnic origin, family structure, history of family physical and psychological health, socioeconomic status, and contact details.

### Research design

A nested cohort design (Murray et al., 2004) with two fixed factors and one random factor was employed. The between-subjects fixed factor was group (intervention, control) and between-subjects random factor was group with two schools nested within each of the two conditions. The within-subject fixed factors were time (2 levels; pre-test and 30 month follow-up).

### Intervention

The Aussie Optimism Positive Thinking Skills Program (AOPTP) is designed to meet the developmental needs of primary school children in years 4 and 5 (equating to ages of approximately 8–10 years). The program includes 10 weekly sessions each lasting 60 min, based on cognitive and behavioral principles, theories, and strategies outlined by Gillham et al. (1995). The program presents age appropriate adaptations of activities used in the Aussie Optimism Program (Roberts et al., 2003). The program included a teacher manual to help with the teaching processes and learning outcomes. The teacher manual includes implementation notes, classroom activities, and all resources needed to help conduct these activities. Student workbooks with worksheets and all information required was provided to the student participants.

The initial session of the AOPTP focuses on confidentiality and group rules. Throughout the program, cognitive and behavioral skills are taught through games and activities. Sessions include; identifying thoughts and feelings; exploring the connection between thoughts, feelings and behavior; evaluating and challenging thoughts; learning to think more accurately and positively; and relaxation and distraction; along with the scheduling of pleasurable events and constructing a fear hierarchy. The program was implemented by classroom teachers who were trained in the AOPTP.

### Procedure

Schools in low socio-economic status (SES) areas from the Canning and Swan School districts in Perth, Western Australia, were randomly selected from the WA Department of Education and Training School Database. These schools were among the largest (top 50%) and poorest (bottom 30%) in the database. A control school was matched for each intervention school in terms of district, SES, class size, and school size.

Informed consent was obtained from schools, parents and participants. Twenty-four schools were approached and 22 agreed to participate. Active and passive consent was sought from children and parents after the study was explained to them via consent and information forms, obtained after the first information session. To increase participation rates, additional consent and information forms were sent to non-respondents 1 week after seeking passive consent. Henry et al. (2002) have found that to increase the representativeness of the sample, passive consent should be sought as children whose parents do not respond to active consent maybe at greater risk of mental health problems than those who do respond to active consent procedures. All available students participated in the universal program as part of their regular health education class.

Trained research assistants provided the assessment battery to children in groups, while the teacher remained present to ensure duty of care was maintained for the children. All groups were introduced to the testing procedure using standard protocols. Children were informed that they could withdraw at any time without penalty, and that their information would be kept confidential unless their responses indicated they were currently distressed, in which case their parents would be confidentially informed in order to seek help for the child. Research assistants read the questions aloud to all class groups, with the assessment battery taking between 30 to 45 min to complete.

Each group facilitator received 8 h of training covering childhood anxiety and depression from the Aussie Optimism Team. Groups were facilitated by Year 4 and 5 teachers who received the teacher's resource that included the content and rationale for all activities, demonstration of the activities, and the provision of opportunities for teachers to practice implementation skills. Teachers facilitating the program received supervision and support from the program developers. Intervention sessions were run weekly with the whole class in their usual classroom at designated times that were convenient to each school over a 10-week period. Implementation of the program was assessed by teacher logs, random sample of children's workbooks and through interviews conducted at the end of the 10-week program.

Facilitators completed checklists to monitor program integrity (88.46% completed the checks), and 25% of each teacher sessions were randomly selected and observed by trained research assistants. Integrity data checks showed that on average, teachers completed 77.5% of the module content. Facilitators reported the mean percentage of content covered for the 10 sessions was 95.61% (*SD* = 5.31%). Attendance rates showed that each student completed on average 9 sessions (*M* = 9.03, *SD* = 2.143).

### Data analysis

Intention to treat analyses were conducted where all students were analyzed in the groups to which they were randomly assigned, regardless of whether students received their allocated treatment, dropped out of the study, or crossed over to another group. Missing data were replaced using the last observation carried forward (LOCF) method. This method fills the missing value with the last available non-missing value of the same subject and is the most popular method for replacing missing values across time.

Stata 10 (StataCorp, 2007) was used to fit regression models for each of the student and parent reported outcomes. For regression models, intra-school clustering on the outcome was controlled by using a sandwich estimator for the standard errors (Rabe-Hesketh and Skrondal, 2005); the independent variable (group) was entered simultaneously with the covariate (the pre-test scores on the outcome).

## Results

### Student attrition

There were 910 students at pre-test, of which 542 responded at 30 month follow-up (a 40.44% attrition rate). Attrition rates did not differ significantly between groups. The analyses of student-reported outcomes were all conducted on the pre-test sample of 910 students.

### Parent attrition

The pre-test parent sample comprised of 572 parents, of which 244 responded at 30 month follow-up (a 57.35% attrition rate). Attrition rates did not differ significantly between groups. The analyses of parent-reported outcomes were all conducted on the pre-test sample of 572 parents.

### Primary outcome analyses

After controlling for pre-test CDI scores, no significant group effects (intervention vs. control) were found for depression at 30 month follow-up (*sr*^2^ × 100 = 0.118, *p* = 0.056). There were also no significant group effects for anxiety at 30 month follow-up (*sr*^2^ × 100 = 0.010, *p* = 0.831). Table 1 shows the mean scores for both groups declined over time within the normal range on the CDI and the SCAS. The mean and standard deviations for student-reported (CDI, SCAS, and CASQ) outcomes at pre-test and 30 month follow-up are presented in Table 1.

**Table 1:** **Means and standard deviations for the student-reported outcomes (*N* = 910)**.

**Outcome**	**Intervention group (*N* = 467)**		**Control group (*N* = 443)**	
	**Mean**	***SD***	**Mean**	***SD***
Pre-test CDI	10.88	9.172	11.5	8.669
Thirty months CDI	6.08	6.491	8.06	8.778
Pre-test SCAS	31.43	19.962	30.6	17.501
Thirty months SCAS	18.99	14.026	19.11	12.09
Pre-test CASQ-P	14.13	3.141	13.8	2.979
Thirty month CASQ-P	14.03	2.890	13.79	2.833
Pre-test CASQ-N	8.26	3.300	8.31	3.350
Thirty month CASQ-N	12.31	3.249	12.06	2.704
Pre-test CASQ Total	5.87	5.297	5.49	5.236
Thirty month CASQ Total	2.07	4.381	1.92	3.859

The results of the regression analysis for the student-reported outcomes at pre-test and 30 month follow-up as measured by the CDI, SCAS, and CASQ are presented in Table 2. As part of the regression analysis, regression coefficients, 95% confidence intervals, robust standard error, part correlations, and *p*-values are presented. There were no significant group effects.

**Table 2:** **Results for regression analysis testing intervention effects on student-reported outcomes (controlling for clustering in 22 schools)**.

**Regression model**	**Regression coefficient (*B*)**	**95% confidence Interval**	**Robust standard error**	**Part-correlation (*sr*)**	***p*-value**
**THIRTY MONTH CDI (DV)**					
Pre-test CDI (covariate)	0.254	(0.183, 0.324)	0.033	0.295	<0.001
Group (IV)	1.817	(−0.047, 3.683)	0.897	0.118	0.056
**THIRTY MONTH SCAS (DV)**					
Pre-test SCAS (covariate)	0.189	(0.136, 0.243)	0.025	0.272	<0.001
Group (IV)	0.275	(−2.370, 2.921)	1.272	0.010	0.831
**THIRTY MONTH CASQ-P (DV)**					
Pre-test CASQ-P (covariate)	0.004	(−0.082, 0.091)	0.041	0.004	0.922
Group (IV)	−0.242	(−0.725, 0.240)	0.232	−0.042	0.3008
**THIRTY MONTH CASQ-N (DV)**					
Pre-test CASQ-N (covariate)	0.029	(−0.059, 0.118)	0.042	0.032	0.499
Group (IV)	−0.246	(−0.735, 0.243)	0.235	−0.041	0.308
**THIRTY MONTH CASQ-TOTAL (DV)**					
Pre-test CASQ-Total (covariate)	0.057	(−0.029, 0.143)	0.041	0.073	0.183
Group (IV)	−0.129	(−0.945, 0.685)	0.392	−0.016	0.744

As measured by the SDQ, after controlling for pre-test hyperactivity scores, group membership (intervention vs. control) predicted a significant 1.21% of the variance in hyperactivity scores at the 30-month follow-up (*sr*^2^ × 100 = 1.21, *p* = 0.012). There was no significant difference for conduct at 30 month follow-up (*sr*^2^ × 100 = 0.025, *p* = 0.511).

Parents reported no significant differences of child internalizing problems. There were no significant group effects for emotion (*sr*^2^ × 100 = 0.028, *p* = 0.567) and peer problems (*sr*^2^ × 100 = −0.057, *p* = 0.360) at 30 month follow-up. There were also no significant group effects for pro-social (*sr*^2^ × 100 = 0.001, *p* = 0.977) and total scores (*sr*^2^ × 100 = 0.046, *p* = 0.244). Table 3 show the means and standard deviations for intervention and control groups on the parent-reported internalizing and externalizing outcomes. As measured by the SDQ, parent-reported emotion, conduct, hyperactivity, peer, prosocial, and total SDQ scores at pre-test and 30 month follow-up are presented.

**Table 3:** **Means and standard deviations for the parent-reported outcomes (*N* = 572)**.

**Outcome**	**Intervention group**		**Control group**	
	**Mean**	***SD***	**Mean**	***SD***
Pre-test emotion	2.51	2.13	2.4	7.00
Thirty month emotion	6.94	2.16	2.29	1.96
Pre-test conduct	1.92	1.77	1.96	1.96
Thirty month conduct	6.52	1.67	6.62	1.73
Pre-test hyperactivity	3.91	2.39	3.48	8.19
Thirty month hyperactivity	7.95	2.18	2.46	2.38
Pre-test peer	2.00	1.65	1.88	1.84
Thirty month peer	6.57	1.57	6.33	1.59
Pre-test prosocial	7.67	1.77	7.81	1.79
Thirty month prosocial	13.06	1.71	13.13	1.66
Pre-test SDQ total	10.34	6.07	9.73	6.46
Thirty month SDQ total	27.97	5.77	28.15	5.74

The results of the regression analysis for the parent-reported outcomes at pre-test and 30 month follow-up are presented in Table 4. As part of the regression analysis, regression coefficients, 95% confidence intervals, robust standard error, part correlations, and *p*-values are presented. As measured by the SDQ, parents reported children displayed significantly less hyperactive behavior at 30 month follow-up in comparison to pre-test scores.

**Table 4:** **Results for regression analysis testing intervention effects on parent-reported outcomes (controlling for clustering in 22 schools)**.

**Regression model**	**Regression coefficient (*B*)**	**95% confidence interval**	**Robust standard error**	**Part-correlation (*sr*)**	***p*-value**
**THIRTY MONTH EMOTION (DV)**					
Pre-test emotion (covariate)	0.504	(0.397, 0.610)	0.051	0.539	<0.001
Group (IV)	0.114	(−0.293, 0.521)	0.195	0.028	0.567
**THIRTY MONTH CONDUCT (DV)**					
Pre-test conduct (covariate)	0.500	(0.418, 0.581)	0.039	0.548	<0.001
Group (IV)	0.086	(−0.182, 0.355)	0.129	0.025	0.511
**THIRTY MONTH HYPERACTIVITY (DV)**					
Pre-test hyperactivity (covariate)	0.597	(0.501, 0.694)	0.046	0.635	<0.001
Group (IV)	0.501	(0.123, 0.878)	0.181	0.110	0.012
**THIRTY MONTH PEER (DV)**					
Pre-test peer (covariate)	0.441	(0.332, 0.549)	0.052	0.486	<0.001
Group (IV)	−0.181	(−0.585, 0.222)	0.194	−0.057	0.360
**THIRTY MONTH PROSOCIAL (DV)**					
Pre-test prosocial (covariate)	0.479	(0.375, 0.583)	0.050	0.507	<0.001
Group (IV)	0.003	(−0.279, 0.287)	0.136	0.001	0.977
**THIRTY MONTH SDQ TOTAL (DV)**					
Pre-test SDQ total (DV)	0.579	(0.475, 0.682)	0.049	0.629	<0.001
Group (IV)	0.553	(−0.391, 1.46)	0.444	0.046	*0.244*

### Secondary outcomes analyses

After controlling for pre-test positive (CASQ-P) and negative (CASQ-N) attribution scores, group membership did not predict a significant proportion of variance for either attribution at post-assessment. There were no significant group effects for CASQ-P at 30 month follow-up (*sr*^2^ × 100 = −0.042, *p* = 0.308). There were also no significant group effects for CASQ-N at 30 month follow-up (*sr*^2^ × 100 = −0.041, *p* = 0.308). Neither were there significant group effects for CASQ-Total (*sr*^2^ × 100 = −0.016, *p* = 0.244). Table 1 shows the mean scores for CASQ-P, CASQ-N, and CASQ-Total.

## Discussion

This study aimed to determine the effectiveness of the Aussie Optimism: Positive Thinking Skills Program (AOPTP) among 9–10 year old children in Perth, Western Australia. This program was designed to prevent anxiety and depression in 9–10 year old children. The results of this universal randomized control trial indicated that the AOPTP is associated with a significant reduction in hyperactive behaviors for the intervention children at 30 month follow-up. The AOPTP did not significantly decrease either depressive or anxious symptomatology or detect any differences in the positive or negative attribution styles for both intervention and control group at 30 month follow-up.

The decrease of hyperactive behaviors as part of the externalizing symptoms are partially consistent with the post-test and 6-month follow up results of Rooney et al. (2013) study, demonstrating that the intervention had reduced the externalizing symptoms (i.e., emotional difficulties) for children from low socio-economic backgrounds. This result supports that participating in the AOPTP is able to provide a significant reduction in externalizing behavior in immediate to medium term durations for 9–10 year old children. The present findings are consistent with Kam et al. (2004), who found an intervention effect in externalizing behaviors after 24 months. However, in contrast to the present findings, Gilliam et al. (1995) and Kam et al. (2004) found positive intervention effects for internalizing behaviors and depressive symptomatology at 2 year follow-up. Unlike the pre, post, 6 and 18 month follow-up study (Rooney et al., 2013) which showed a prevention effect for depressive symptomatology at post-test, no significant effects for depressive symptomatology was found in the current study, suggesting that further investigation is required to determine whether the program is able to provide a reduction in internalizing symptomatology.

The present study's findings do not support the AOPTP in having medium term benefits in preventing anxiety or depression. Additionally, findings do not support medium term benefits of children who received the intervention from developing a less pessimistic attribution style compared to the children who did not receive the intervention. One of the reasons for not finding an intervention effect at 30 month follow-up may have been because children may have found the intervention too complex and subsequently did not understand the cognitive component of the intervention, thus inhibiting the opportunity for children to internalize the cognitive techniques taught in the intervention. Rooney et al. (2013) stated that future revisions of the AOPTP should simplify the cognitive component, and while the AOPTP that was employed in this study had built on this, it could still potentially be too complex. Additionally, future revisions should emphasize the development of emotional competence, focusing on teaching children skills to address their feelings and empathy, which recent research has suggested is important for children of this age (Buckley et al., 2003; Cole et al., 2007).

In the field of public health, it is well-accepted that small effect sizes can have a considerable impact on the societal burden of mental health, for both internalizing and externalizing disorders (Bolier et al., 2013). As the AOPTP is only a short, 10-week program (that did not have any additional sessions) that is relatively inexpensive, findings that found evidence for a continued impact on externalizing behavior up to 30 months after the program, provides evidence supporting the efficacy of the program. Externalizing problems are associated with a raft of detrimental outcomes that facilitate negative consequences on both the child and society, including conduct problems, school absence and later unemployment and incarceration (Stevenson and Goodman, 2001; Liu, 2004).

Differences in implementing the AOPTP from other universal programs may partly explain why there were no prevention effects at medium term follow-up. For example, one study evaluated the long term effectiveness of the FRIENDS program in preventing anxiety and depression in children (Barrett et al., 2006). Results indicated that reductions in anxiety for intervention group were maintained for Grade 6 students and that the group also reported significantly lower ratings of anxiety at long term (36 month) follow-up. The major differences between Barrett et al. (2006) and Rooney et al. (2013) programs is the use of a trained clinical psychologist to deliver the intervention in a small group of 9–10 children, as seen in Barrett et al. (2006) study. There is no empirical evidence indicating significant differences between using psychologists and teachers as facilitators of universal programs (Barrett and Turner, 2001). However, as psychologists were responsible for the development of these school based universal prevention programs; they may have greater expertise in delivering the material employed by these programs. There has also been no evidence of significant differences between children receiving universal intervention programs in small groups rather than larger groups of children. However, children receiving intervention in small groups may have an advantage of less peer distractions and more personal and privileged contact with the facilitator, thus allowing the facilitator to spend more time answering questions and focusing on difficult concepts.

Future revisions of the AOPTP could explore the benefits of using a psychologist as facilitator. Cost saving is one of the main benefits and attractions for school administrators in using universal rather than selective or indicated programs. However, with psychologists as facilitators, this may defeat any cost savings of a universal program due to the added cost of employing psychologists to facilitate the intervention. Therefore, it will be a matter of exploring the benefits and negatives of employing psychologists and determining whether these outweigh the cost benefits of using teachers as facilitators. While the present study employed a 30-month follow up equating to a medium term effect, future evaluations of the program should examine the long term effects of the program.

The AOPTP could also explore the possibilities of using small intervention groups. While maintaining the program integrity, the mean percentage of content covered by the facilitators will be higher (as it is easier to manage small group of children and more likely the facilitators are able to do all the activities listed in the Teacher Manual) and children may benefit from individualized, attention and more focus to practise on skills taught if the program is offered in a small group format in contrast to larger groups. However, smaller groups of children will be more costly and timely for schools. For example, more facilitators will be required to run the program, extra classrooms are needed to assign the students into number of small groups and a new timetable will have to be designed in order to suit the facilitator's schedule and be convenient to the schools involved.

The AOPTP is a universal cognitive-behavioral strategy developed with the aim of preventing anxiety and depression in school aged children. The program has shown to be effective in preventing depressive symptomatology in the short term and reducing the externalizing behavior at short and medium term (Rooney et al., 2013). However, the 30 month follow-up study did not show longer term effect of internalizing behaviors for 9–10 year old children from low SES backgrounds, although findings suggested the program was beneficial at addressing externalizing behaviors. Future research and revisions of the current AOP-PTS program is required to determine strategies that are more effective over the medium and longer term to address internalizing problems in 9–10 year old children.

### Conflict of interest statement

The authors declare that the research was conducted in the absence of any commercial or financial relationships that could be construed as a potential conflict of interest.
